# Supplementary material for: Case–control study of the association of chronic acid suppression and social determinants of health with COVID-19 infection
Source: Sci Rep. 2021 Oct 25;11:20987. doi: 10.1038/s41598-021-00367-7 (PMC8545937; doi:10.1038/s41598-021-00367-7)
Supplement: Supplementary file 1 — Supplementary Information. [file 41598_2021_367_MOESM1_ESM.pdf]

Title

Case-control Study of the Association of Chronic Acid Suppression and Social Determinants of Health with COVID-19 Infection

Authors

Bing Zhang, MD MAS

Anna L. Silverman, MD

Saroja Bangaru, MD

Douglas Arneson, PhD

Sonya Dasharathy, MD

Nghia Nguyen, MD MAS

Diane Rodden, MD

Jonathan Shih, BS

Atul J. Butte, MD PhD

Wael Noor El-Nachef, MD PhD

Brigid S. Boland, MD

Vivek A. Rudrapatna, MD PhD

Supplementary Fig. S1 - Incorporation of chronic acid suppression use (combined PPI and H2RA) into statistical model

Subgroup	No. of Patients (%)	Odds Ratio	P Value
Overall	861 (100)%		
SARS−CoV2 test month			
Mar	195 (22.6%)	ref	ref
Apr	253 (29.4%)	0.81 (0.74−0.89)	9.18e−06
May	360 (41.8%)	0.67 (0.62−0.74)	0
Jun	53 (6.2%)	0.79 (0.69−0.91)	0.00147
Sex			
Male	397 (46.1%)	ref	ref
Female	464 (53.9%)	0.89 (0.83−0.95)	0.000202
Ethnicity			
Not Latinx	593 (68.9%)	ref	ref
Latinx	189 (22%)	1.25 (1.16−1.36)	3.9e−08
BMI			
< 30	524 (60.9%)	ref	ref
>= 30	227 (26.4%)	1.11 (1.03−1.19)	0.00542
GERD			
No	704 (81.8%)	ref	ref
Yes	154 (17.9%)	0.89 (0.79−1)	0.0509
Heart Failure			
No	823 (95.6%)	ref	ref
Yes	38 (4.4%)	1.14 (0.98−1.33)	0.0933
Stroke			
No	827 (96.1%)	ref	ref
Yes	34 (3.9%)	0.89 (0.75−1.05)	0.163
Solid Tumor			
No	784 (91.1%)	ref	ref
Yes	77 (8.9%)	0.85 (0.76−0.95)	0.00425
Dementia			
No	836 (97.1%)	ref	ref
Yes	25 (2.9%)	1.32 (1.09−1.6)	0.00423
EtOH use disorder			
No	804 (93.4%)	ref	ref
Yes	32 (3.7%)	0.81 (0.69−0.95)	0.00947
Healthcare Worker			
No	654 (76%)	ref	ref
Yes	160 (18.6%)	0.94 (0.87−1.02)	0.129
Social Deprivation Index			
<= 65	509 (59.1%)	ref	ref
> 65	352 (40.9%)	1.04 (0.97−1.11)	0.27
Time spent home			
<= 85.0%	91 (10.6%)	ref	ref
> 85.0%	770 (89.4%)	0.96 (0.86−1.07)	0.441
Public transportation usage			
<= 4.4%	367 (42.6%)	ref	ref
> 4.4%	494 (57.4%)	1.1 (1.03−1.18)	0.00317
Acid Suppression Use			
No	701 (81.4%)	ref	ref
Yes	159 (18.5%)	1.04 (0.92−1.17)	0.515

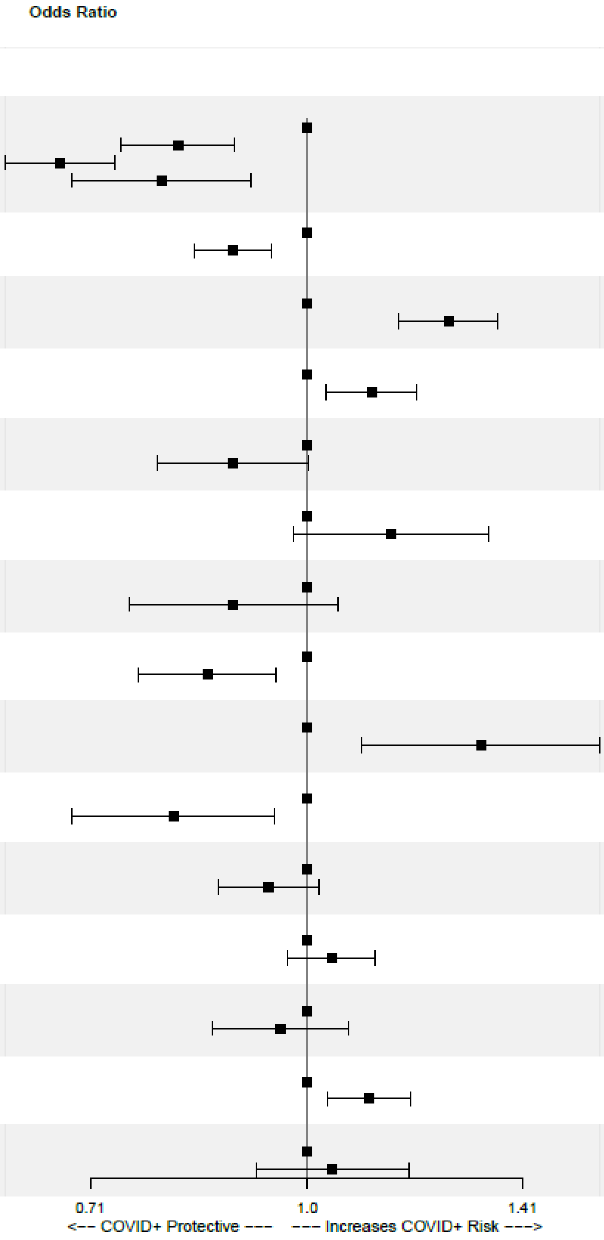

Adjusted odds ratios and P-values were calculated when compared to baseline (ref).

Supplementary Methods: Chart Extraction Variables

1. Location of test: 0 = UCSF, 1 = UCSD, 2 = UCLA
2. Patient MRN: enter MRN (note that this column exists only for internal use and can be deleted prior to transfer of data to UCSF or other site)
3. COVID-19 DX: 0 = no; 1 = yes
4. Date of COVID-19 test: mm/dd/yyyy
5. Age: enter age at the time of the COVID-19 test
6. Sex: 0 = male, 1 = female. If neither of the above leave blank
7. Race: 0 = White, 1 = Black or African American, 2 = American Indian or Alaska Native, 3 = Asian, 4 = Native Hawaiian or Other Pacific Islander
8. Ethnicity: 0 = not Hispanic or Latino; 1 = Hispanic or Latino
9. Zip code: enter zip code
10. Chronic PPI or H2 Blocker (defined as more than 4 weeks): 0 = no; 1 = yes
11. PPI Type: enter the generic name of the PPI that the patient is on at the time of COVID test, or NA if unknown
12. PPI Blocker Dose (mg): enter dose or NA if unknown
13. PPI Frequency: 0 = once daily; 1 = twice daily, NA = unknown
14. Duration of PPI Use (months): enter the total duration of time (in months) with regular use of PPI. If the patient is a PRN user of PPI, annotate as **-1**. If the number of months is unknown but the number of years is documented (e.g. 2 years), report as the equivalent number of months (e.g. 24 months). If the start date of PPI is unclear by chart review annotate as NA
15. H2 Blocker: 0 = no, 1 = yes
16. H2 Blocker Type: type name of H2 blocker that the patient is on at the time of COVID test
17. H2 Blocker Dose (mg): enter number
18. H2 Blocker Frequency: 0 = once daily; 1 = twice daily
19. Duration of H2 Blocker use (months): enter number
20. GERD/Reflux/Regurgitation: 0 = no; 1 = yes

CCI

21. MI (anytime in the past prior to COVID Test): 0 = no; 1 = yes. CAD in the absence of myocardial death (defined by EKG, Echocardiography, PET, MRI or equivalent) should NOT be treated as evidence of an MI. Impaired myocardial function (e.g. wall motion abnormalities) may be treated as a 'yes' for this category even if reversible ischemia has not been clearly established.
22. CHF (anytime in the past prior to COVID Test): 0 = no; 1 = yes. This may include conditions with structural heart damage commonly characterized by decreased ejection fraction but may also include conditions with persevered ejection fraction (i.e. 'diastolic heart failure'). Many cardiomyopathies will fall under this category.
23. Peripheral Vascular Disease (anytime in the past prior to COVID Test): 0 = no; 1 = yes. These include peripheral arterial disease and as well as venous insufficiency.
24. Stroke (CVA or TIA) (anytime in the past prior to COVID Test): 0 = no; 1 = yes. These encompass both ischemic and hemorrhagic varieties, and overall reflect an increased risk of pneumonia due to their association with dysphagia and aspiration.
25. Dementia (anytime in the past prior to COVID Test): 0 = no; 1 = yes
26. COPD (anytime in the past prior to COVID Test): 0 = no; 1 = yes
27. Connective Tissue Disease (anytime in the past prior to COVID Test): 0 = no; 1 = yes. These generally refer to autoimmune conditions of the skin, joints, and soft tissue but may encompass other entities. If edge cases arise they can be discussed and adjudicated in group meetings.
28. Peptic Ulcer Disease: 0 = no; 1 = yes. Patients for whom these ulcers have resolved (e.g. NSAID discontinuation, H Pylori treatment with confirmation of clearance) can be treated as 'no'.
29. Liver Disease: 0 = no; 1 = yes. Self-limited or resolved liver diseases (e.g. Hep A, Drug-induced liver injury) should not be treated as ongoing liver disease.
30. DM (1 or 2): 0 = none or diet-controlled, 1 = uncomplicated, 2 = end-organ damage
31. Hemiplegia (anytime in the past prior to COVID Test): 0 = no; 1 = yes

32. Creatinine ≥ 3, dialysis, s/p kidney transplant, uremia (most recent lab value prior to COVID test): 0 = no; 1 = yes
33. Solid Tumor: 0 = none, 1 = localized, 2 = metastatic
34. Leukemia: 0 = no; 1 = yes
35. Lymphoma: 0 = no; 1 = yes
36. AIDS: 0 = no; 1 = yes, NA if unknown. Annotate based on the most recent CD4 count of the patient if available within the 1y prior to a COVID-19 test, with AIDS defined as the presence of HIV positivity (by PCR or serologies) with an absolute CD4 count less than 200.
37. CCI Score: calculated from above using - https://www.mdcalc.com/charlson-comorbidity-index-cci

Other Comorbidities

38. HTN (anytime in the past prior to COVID Test): 0 = no; 1 = yes. HTN should be clearly noted by a primary care note. At the reviewer's discretion, this diagnosis may be ascertained in the setting of repeated elevations in ambulatory blood pressure (>140/90). Missing data should be rare for this category.
39. Asthma: 0 = no, 1 = yes.
40. HIV: 0 = no, 1 = HIV, 2 = AIDS.
41. CKD (Cre < 3): 0 = no, 1 = yes.
42. Medications: immunocompromised medications.
43. BMI: enter number. If BMI as captured within the last year prior to COVID19 testing is not explicitly reported in the EHR, but weight is, use the following website. Treat height as a fixed variable (ok to use old height measurements) https://www.nhlbi.nih.gov/health/educational/lose_wt/BMI/bmicalc.htm
44. IBD (anytime in the past prior to COVID Test): 0 = no; 1 = yes
45. Autoimmune disease (anytime in the past prior to COVID Test): 0 = no; 1 = yes
46. Hx Cancer (anytime in the past prior to COVID Test): 0 = no; 1 = yes. These typically will encompass both solid and hematological malignancies. Patients who have had cutaneous malignancies (e.g. basal cell cancer) which have been excised and deemed cured may be treated as a 0. Other malignancies that were identified and definitively resected at an early stage without need for systemic chemotherapy may be discussed in group with the possibility of revising this variable: it is intended to capture states of immunocompromise related to history of cancer treatment.
47. Type of cancer: free text.
48. Prior cancer Tx: 1 = chemo (including immunotherapy); 2 = bone marrow transplant, 3 = other
49. Chronic Opiate Use: 0 = no; 1 = yes
50. Immunocompromised (by med or comorbidity): 0 = no, 1 = yes
51. Recent antibiotic use (< 3mos): 0 = no; 1 = yes
52. Smoker (including marijuana): 0 = none, 1 = prior, 2 = current, NA if missing
53. EtOH use disorder: 0 = none, 1 = prior, 2 = current, NA if missing
54. Healthcare worker: 0 = no, 1 = yes
55. Group Living (SNF, rehab, shelter, etc): 0 = no; 1 = yes, NA if missing
56. Apartment: 0 = no; 1 = yes

Supplementary Methods: Discussion on Coding of Specific Variables

1. Autoimmune diseases – IBD, ankylosing spondylitis, myasthenia gravis, type 1 diabetes, rheumatoid arthritis; hypothyroidism is not charted as autoimmune
2. Every other day PPI – code as PRN
3. Immunosuppression medications – steroids, biologics, small molecules, chemotherapy, MTX/6MP
4. Diabetes and cirrhosis should be considered as immunocompromised
5. For CCI – current solid tumors are recorded as yes
6. Basal cell cancer – not counted as solid cancer
7. The most recent PPI should be recorded on datasheet
8. MI – counted if there is history of NSTEMI or STEMI

Supplementary Table 1 – The STROBE guideline for case-control studies

STROBE Statement—Checklist of items that should be included in reports of ***case-control studies***

	Item No	Recommendation	Page No
**Title and abstract**	1	(*a*) Indicate the study's design with a commonly used term in the title or the abstract	1
		(*b*) Provide in the abstract an informative and balanced summary of what was done and what was found	3
**Introduction**			
Background/rationale	2	Explain the scientific background and rationale for the investigation being reported	4
Objectives	3	State specific objectives, including any prespecified hypotheses	4
**Methods**			
Study design	4	Present key elements of study design early in the paper	8
Setting	5	Describe the setting, locations, and relevant dates, including periods of recruitment, exposure, follow-up, and data collection	8-9
Participants	6	(*a*) Give the eligibility criteria, and the sources and methods of case ascertainment and control selection. Give the rationale for the choice of cases and controls	9
		(*b*) For matched studies, give matching criteria and the number of controls per case	9
Variables	7	Clearly define all outcomes, exposures, predictors, potential confounders, and effect modifiers. Give diagnostic criteria, if applicable	9-11
Data sources/ measurement	8*	For each variable of interest, give sources of data and details of methods of assessment (measurement). Describe comparability of assessment methods if there is more than one group	9-11
Bias	9	Describe any efforts to address potential sources of bias	7, 9-11
Study size	10	Explain how the study size was arrived at	8-9
Quantitative variables	11	Explain how quantitative variables were handled in the analyses. If applicable, describe which groupings were chosen and why	8-11
Statistical methods	12	(*a*) Describe all statistical methods, including those used to control for confounding	8-11
		(*b*) Describe any methods used to examine subgroups and interactions	8-11
		(*c*) Explain how missing data were addressed	8-11
		(*d*) If applicable, explain how matching of cases and controls was addressed	8-11
		(*e*) Describe any sensitivity analyses	N/A
**Results**			
Participants	13*	(a) Report numbers of individuals at each stage of study—eg numbers potentially eligible, examined for eligibility, confirmed eligible, included in the study, completing follow-up, and analysed	4
		(b) Give reasons for non-participation at each stage	N/A
		(c) Consider use of a flow diagram	Fig 1
Descriptive data	14*	(a) Give characteristics of study participants (eg demographic, clinical, social) and information on exposures and potential confounders	Fig 2
		(b) Indicate number of participants with missing data for each variable of interest	4
Outcome data	15*	Report numbers in each exposure category, or summary measures of exposure	Fig 2

Main results	16	(*a*) Give unadjusted estimates and, if applicable, confounder-adjusted estimates and their precision (eg, 95% confidence interval). Make clear which confounders were adjusted for and why they were included	5, Fig 3
		(*b*) Report category boundaries when continuous variables were categorized	5, Fig 3
		(*c*) If relevant, consider translating estimates of relative risk into absolute risk for a meaningful time period	N/A
Other analyses	17	Report other analyses done—eg analyses of subgroups and interactions, and sensitivity analyses	5-6, Fig 3
**Discussion**			
Key results	18	Summarise key results with reference to study objectives	6-7
Limitations	19	Discuss limitations of the study, taking into account sources of potential bias or imprecision. Discuss both direction and magnitude of any potential bias	7-8
Interpretation	20	Give a cautious overall interpretation of results considering objectives, limitations, multiplicity of analyses, results from similar studies, and other relevant evidence	7-8
Generalisability	21	Discuss the generalisability (external validity) of the study results	8
**Other information**			
Funding	22	Give the source of funding and the role of the funders for the present study and, if applicable, for the original study on which the present article is based	17-18

*Give information separately for cases and controls.

Note: An Explanation and Elaboration article discusses each checklist item and gives methodological background and published examples of transparent reporting. The STROBE checklist is best used in conjunction with this article (freely available on the Web sites of PLoS Medicine at http://www.plosmedicine.org/, Annals of Internal Medicine at http://www.annals.org/, and Epidemiology at http://www.epidem.com/). Information on the STROBE Initiative is available at http://www.strobe-statement.org.

Supplementary Table 2 – Features included in logistics regression model

Location (Site)
Date of COVID-19 Test
Age
Sex
Race
Ethnicity
PPI Use
H2RA Use
GERD/Reflux
Myocardial Infarction
Congestive Heart Failure
Peripheral Vascular Disease
Stroke
Dementia
COPD
Connective Tissue Disease
Peptic Ulcer Disease
Liver Disease
Diabetes Mellitus
Hemiplegia
Moderate to Severe CKD
Solid Tumor
Leukemia
Lymphoma
AIDS
Hypertension
Asthma
HIV
CKD (Cre < 3)
BMI
IBD
Autoimmune
Hx Cancer
Chronic opiate use
Immunocompromised (by med or comorbidity)
Smoker (including marijuana)
EtOH use disorder
Healthcare worker
Apartment (living situation)
CCI

Acid Suppression Use
Median Income (Zip code - US Census)
Public Transportation (Zip code - US Census)
Walk to Work (Zip code - US Census)
Population Density (Zip code - US Census)
Housing Density (Zip code - US Census)
Percent Low Risk Factors (Zip code - Community Resilience Estimates)
Percent Moderate Risk Factors (Zip code - Community Resilience Estimates)
Percent High Risk Factors (Zip code - Community Resilience Estimates)
Mask Use: Never (County - New York Times)
Mask Use: Rarely or More Often (County - New York Times)
Mask Use: Sometimes or More Often (County - New York Times)
Mask Use: Frequently or More Often (County - New York Times)
Mask Use: Always (County - New York Times)
Time Spent At Retail Stores (County - Google; as a percentage of baseline)
Time Spent At Grocery Stores (County - Google; as a percentage of baseline)
Time Spent At Parks (County - Google; as a percentage of baseline)
Time Spent At Public Transit (County - Google; as a percentage of baseline)
Time Spent At Workplaces (County - Google; as a percentage of baseline)
Time Spent At Residential Areas (County - Google; as a percentage of baseline)
Social Deprivation Index (Zip code)
Median Percentage of Time Spent Home (Zip code - SafeGraph)

Supplementary Table 3 – Chi-squared and ANOVA tests for associations between all variables and positivity for SARS-CoV2

Covariate	Test	P-value	P-adjusted
Testing Site	Chi-squared test	0.942012219	1
Testing Date	ANOVA	5.16E-16	3.20E-14
Age	ANOVA	0.076437113	1
Sex	Chi-squared test	0.00154644	0.095879295
Race	Chi-squared test	0.201009686	1
Ethnicity	Chi-squared test	1.06E-08	6.59E-07
Proton Pump Inhibitors Use	Chi-squared test	0.148170373	1
H2 Receptor Antagonists Use	Chi-squared test	0.748249482	1
Gastroesophageal Reflux Disease	Chi-squared test	0.047452076	1
Myocardial Infarction	Chi-squared test	1	1
Congestive Heart Failure	Chi-squared test	0.386352554	1
Peripheral Vascular Disease	Chi-squared test	0.983167162	1
Stroke	Chi-squared test	0.623834584	1
Dementia	Chi-squared test	0.098281611	1
Chronic Obstructive Pulmonary Disease	Chi-squared test	0.818699167	1
Connective Tissue Disease	Chi-squared test	0.693219511	1
Peptic Ulcer Disease	Chi-squared test	0.734827398	1
Liver Disease	Chi-squared test	0.284374783	1
Diabetes Mellitus	Chi-squared test	0.07966939	1
Hemiplegia	Chi-squared test	0.987691116	1
Moderate to Severe Chronic Kidney Disease	Chi-squared test	0.865733418	1
Solid Tumor	Chi-squared test	0.002275338	0.14107093
Leukemia	Chi-squared test	1	1
Lymphoma	Chi-squared test	1	1

AIDS	Chi-squared test	0.986026826	1
Hypertension	Chi-squared test	0.584827925	1
Asthma	Chi-squared test	0.859351854	1
HIV	Chi-squared test	0.06262942	1
Mild Chronic Kidney Disease	Chi-squared test	0.488523466	1
BMI	ANOVA	9.40E-04	0.058251806
Irritable Bowl Disease	Chi-squared test	1	1
Autoimmune	Chi-squared test	0.795563529	1
History of Cancer	Chi-squared test	0.00711169	0.440924784
Chronic Opiate Use	Chi-squared test	0.070603134	1
Immunocompromised	Chi-squared test	0.89905475	1
Smoker	Chi-squared test	0.713019887	1
EtOH Use Disorder	Chi-squared test	0.217114728	1
Healthcare Worker	Chi-squared test	0.1429991	1
Apartment	Chi-squared test	0.031996071	1
Charlson Comorbidity Index	ANOVA	0.296930111	1
Acid Suppression Use	Chi-squared test	0.097035881	1
Median Household Income - Census	Chi-squared test	0.363997104	1
Public Transportation Usage - Census	Chi-squared test	0.129609183	1
Walk to Work - Census	Chi-squared test	0.665262927	1
Population Density - Census	Chi-squared test	0.314843982	1
Housing Density - Census	Chi-squared test	0.994148826	1
Low Risk Factors - Community Resilience Estimates	Chi-squared test	4.45E-04	0.027584464
Moderate Risk Factors - Community Resilience Estimates	Chi-squared test	0.010628288	0.658953885
High Risk Factors - Community Resilience Estimates	Chi-squared test	0.011575204	0.717662625
Mask Use Never - New York Times	Chi-squared test	0.006267572	0.388589466
Mask Use Rarely - New York Times	Chi-squared test	0.017956826	1
Mask Use Sometimes - New York Times	Chi-squared test	0.25226316	1

Mask Use Frequently - New York Times	Chi-squared test	0.022423814	1
Mask Use Always - New York Times	Chi-squared test	0.005805974	0.359970372
Time Spent at Retail Locations - Google	Chi-squared test	0.07022619	1
Time Spent at Grocery Locations - Google	Chi-squared test	0.543140492	1
Time Spent at Parks Locations - Google	Chi-squared test	6.34E-04	0.039301482
Time Spent at Transit Locations - Google	Chi-squared test	0.678044176	1
Time Spent at Workplace Locations - Google	Chi-squared test	0.395108117	1
Time Spent at Residential Locations - Google	Chi-squared test	0.619551444	1
Social Deprivation Index	Chi-squared test	0.011227352	0.696095804
Median Percentage Time Spent at Home - SafeGraph	Chi-squared test	0.005181477	0.321251564
